# Different Therapeutic Effects of CO_2_ and Diode Laser Irradiation on Tooth Movement-Related Pain

**DOI:** 10.3389/fneur.2020.00481

**Published:** 2020-06-05

**Authors:** Takako Tsuchiya, Naoya Hasegawa, Misato Yugawa, Au Sasaki, Naoto Suda, Kazunori Adachi

**Affiliations:** ^1^Division of Orthodontics, Meikai University School of Dentistry, Sakado, Japan; ^2^Division of Pharmacology, Meikai University School of Dentistry, Sakado, Japan

**Keywords:** jaw-opening reflex, orthodontic pain, low-level laser therapy, CO_2_ laser, diode laser

## Abstract

Although orthodontic treatment is common, orthodontic force often induced pain. Low-level laser therapy (LLLT) has been investigated to improve therapeutic comfort. In dentistry, LLLT is mainly applied using two types of lasers, CO_2_ and diode lasers, whose biological actions are thought to be associated with wavelength (CO_2_: 10,600 nm; diode: 808 nm). The analgesic effect of LLLT on orthodontic treatment-related pain is widely reported but inconsistent. This study aimed to (1) determine whether irradiation with a CO_2_ or diode laser attenuates orthodontic treatment-related pain using the jaw-opening reflex model, (2) elucidate the optimal irradiation protocol for both lasers to obtain the maximal analgesic effect, (3) evaluate the effects of laser irradiation on other biological features [e.g., tooth movement, glial fibrillary acidic protein (GFAP) expression, and temperature alterations] and (4) investigate the mechanism underlying the analgesic effect of laser irradiation. In this animal model, orthodontic treatment-induced pain manifested as a significantly reduced the threshold for inducing the jaw-opening reflex on the orthodontically treated side compared with the contralateral side. GFAP expression in the bilateral trigeminal ganglia (TGs) was significantly increased by the application of orthodontic force. CO_2_ laser irradiation of the orthodontically treated region significantly increased the threshold for inducing the jaw-opening reflex and the peripheral temperature. Similar reductions in jaw-opening reflex excitability were induced by surface anesthesia and thermal stimulation but not, the diode laser. Neither CO_2_ nor diode laser irradiation altered GFAP expression in the TGs. Infiltration anesthesia also significantly increased the threshold for inducing the jaw-opening reflex on each anesthetized side. Irradiation (30 s) by either laser immediately after orthodontic force application (preirradiation) significantly decreased jaw-opening reflex excitability and GFAP expression in the bilateral TGs the next day. However, thermal stimulation immediately after orthodontic force application failed to alter jaw-opening reflex excitability the next day. Laser irradiation did not alter tooth movement; however, an optimized irradiation protocol for aiding tooth movement is suggested. In conclusion, both CO_2_ and diode lasers are able to prevent orthodontic treatment-related pain. Furthermore, the involvement of temperature alterations and surface anesthesia in the analgesic effect induced by CO_2_ laser irradiation is suggested.

## Introduction

Orthodontic force-induced pain, which appears within a day and lasts up to a week after the placement or reactivation of orthodontic force, is frequently observed in patients (1–4). Although inflammation in the periodontium is required to achieve accurate tooth movement (5, 6), pain sensation is an adverse effect for patients (1). In dentistry, acidic non-steroidal anti-inflammatory drugs (NSAIDs) are frequently used for severe orofacial pain (e.g., trauma caused by tooth extraction); however, acidic NSAIDs are not recommended for orthodontic patients because prostaglandin E_2_, the eicosanoid that is inhibited by acidic NSAIDs, mediates the maturation of osteoclasts (7–10). To protect patients from this disadvantage of orthodontic therapy, alternative analgesic methods have been investigated, and low-level laser therapy (LLLT) is one of these approaches (11–15). Several types of lasers are available for therapeutic purposes and are classified based on wavelength (16, 17). Lasers in the near-infrared (700–1,200 nm) range (e.g., diode and Nd:YAG lasers) are able to penetrate surface tissue and reach deep tissue. On the other hand, lasers in the far-infrared (>1,200 nm) range (e.g., CO_2_ lasers) are absorbed in the surface tissue and increase the tissue temperature. Despite these differences, both types of lasers have a photodynamic effect (e.g., photons activate mitochondria) (16, 17), which has been thought to be the basis of the therapeutic effect of laser irradiation. LLLT has been applied to treat temporomandibular disorder (18) and endodontic and periodontal diseases (17) in dentistry, and an analgesic effect of LLLT on orthodontic force-induced pain has been reported, although not consistently (11, 12, 19, 20). To evaluate the analgesic effect quantitatively and to elucidate the appropriate CO_2_ or diode laser irradiation strategy for orthodontic force-induced pain, the changes in jaw-opening reflex excitability caused by laser irradiation were measured in the experimental tooth movement (ETM) animal model. In this animal model, the application of orthodontic force increases jaw-opening reflex excitability, which is inhibited by the repetitive administration of acidic NSAIDs (e.g., aspirin) (21). This result indicates that orthodontic force induces peripheral inflammation. Recently, a relationship between the presence of peripheral inflammation and morphological alterations in satellite glial cells (SGCs) was reported (22). In the trigeminal ganglia (TGs), SGCs surround the somata of neurons (23) and are excited by peripheral injury (22, 24) and/or inflammation (23, 25). Since the excitation of SGCs was evaluated by an increase in the expression of glial fibrillary acidic protein (GFAP) in those reports, the effect of laser irradiation on GFAP expression in the TGs was examined. Moreover, the distance of ETM was measured to evaluate the effects (adverse or beneficial) of laser irradiation on the maturation of osteoclasts indirectly. Finally, the mechanism underlying the analgesic effect of laser irradiation was investigated by applying thermal stimulation and local anesthesia.

## Materials and Methods

### Animals and Experimental Design

The experimental procedure in this study was approved by the Animal Experimentation Committee of Meikai University School of Dentistry. The animal treatments were performed in accordance with the Animal Research: Reporting of *In Vivo* Experiments (ARRIVE) guidelines and the institutional guidelines for the care and use of experimental animals described in the US National Institutes of Health's *Guide for the Care and Use of Laboratory Animals*. One hundred seventy-six 10-week-old male Wistar rats (273 ± 2.7 g, Sankyo Laboratory Service, Tokyo, Japan) were used in this study. The rats were maintained in a temperature-controlled (23 ± 1°C) environment under a 12 h light-dark cycle with free access to food and water. After the application of orthodontic force, the food was replaced with ground chow (MF, Oriental Yeast Co., Tokyo). The rats were divided into the intact and ETM groups (*n* = 8 each). Jaw-opening reflex excitability in the intact group was evaluated as an acute experiment, whereas jaw-opening reflex excitability in the ETM group was assessed at one (D1), three (D3), or seven (D7) days after the application of orthodontic force. Thirty or 600 s of CO_2_ or diode laser irradiation was applied in the intact and D1 groups. Additional D1 animals received 15 s of CO_2_ laser irradiation or 30 s of guide laser irradiation, local anesthesia (infiltration or surface) or thermal stimulation before the evaluation of jaw-opening reflex excitability. Thirty seconds of CO_2_ or diode laser irradiation was applied immediately after the application of orthodontic force (preirradiation: PI) in another set of animals, and jaw-opening reflex excitability was evaluated one (PI-D1), three (PI-D3), or seven (PI-D7) days after the application of orthodontic force for comparison with that in the D1, D3, and D7 groups. Thermal stimulation was also applied in another set of animals immediately after the application of orthodontic force (preheating: PH), and jaw-opening reflex excitability was evaluated the next day.

### Application of Experimental Orthodontic Force and Evaluation of Jaw-Opening Reflex Excitability

**Figure 1:**
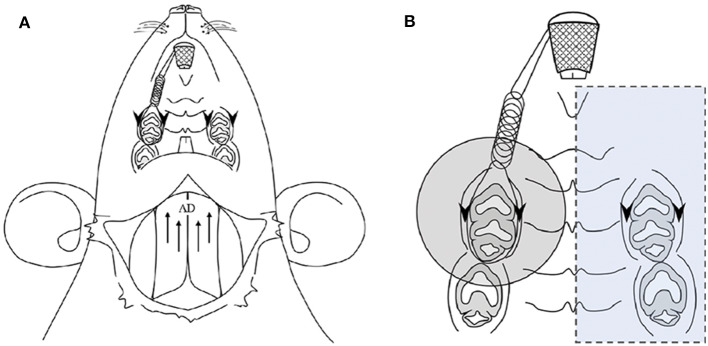
Application of experimental orthodontic force and recording of the jaw-opening reflex **(A)** and the oral region targeted for laser irradiation **(B)**. **(A)** Schematic drawing of the application of experimental orthodontic force to the right maxillary first molar by the placement of a nickel-titanium closed-coil spring between the incisors. Electrodes were placed for recording the bilateral anterior digastric (AD) muscles by electromyography (EMG) as well as for electrical stimulation of the gingiva of the bilateral maxillary first molars. **(B)** The region targeted for laser irradiation is indicated by a shaded circle (ϕ14.6 mm, 1.67 cm^2^). The left molars and gingiva were covered with a silicone rubber impression material to avoid laser irradiation (blue square). Arrows: electrode insertion regions for EMG recording of AD muscles. Arrowheads: electrode insertion regions for electrical stimulation.

An orthodontic apparatus was applied in every group except the intact group. After anesthetization with isoflurane (3.0%, 1.0 L/min), a closed-coil titanium-nickel spring (855–180; American Orthodontics, WI, USA) was placed between the maxillary incisors and the right first molar for continuous application of orthodontic force (Figure 1A). The right first molar was ligated by a wire (0.08 in. 506-01, Tommy, Tokyo, Japan), and the incisors were bonded to a mesh sheet (110-00, Tommy) by light-cured dental adhesive resin cement (Optiband Ultra 740-0293 KaVo Dental Systems Japan Co., Ltd., Tokyo, Japan). The force magnitude was confirmed by a tension gauge (DTN-150, Teclock, Tokyo, Japan), and the spring elongation was ~6 mm to obtain continuous orthodontic force (50 g) (21). For the evaluation of jaw-opening reflex excitability, the animals were anesthetized with isoflurane and underwent tracheal intubation. During surgery, the concentration of isoflurane was maintained at 2.0% (1.0 L/min) to eliminate expression of the nocifensive reflex. Pairs of Teflon^®^-insulated stainless-steel wires (40 gauge; Cooner wire, Chatsworth, CA, USA) were implanted to record the heart rate and the activity of the bilateral anterior digastric muscles by electromyography (EMG). Another pair of electrodes was placed in the buccal and palatal gingiva of the bilateral maxillary first molars for local electrical stimulation (21, 26). After surgery, the concentration of isoflurane was reduced (<1.25%, 1.0 L/min) until the toe-pinch reflex was detected. The animal's body temperature and heart rate were maintained at physiological levels of ~37°C and 330–430 beats/min, respectively. Passing currents (200 μs) were ascendingly applied to the right and then to the left maxillary first molar region to evoke anterior digastric muscle activity, which was defined as the jaw-opening reflex when it exceeded the baseline muscle activity (mean amplitude of 100-ms duration before stimulation + 5 SD) with constant latency. The current stimulation intensity that induced anterior digastric muscle activity in more than three out of five trials was defined as the jaw-opening reflex threshold. Then, suprathreshold (×1.5, ×2, and ×3) stimulation was applied. The data were stored (6 kHz; CED 1401 Plus; Cambridge Electronic Design, Cambridge, UK) to analyze the latency, duration, and area under the curve (AUC) of the jaw-opening reflex (Spike2 ver. 7; Cambridge Electronic Design). In all experimental groups, excluding the preirradiation groups, jaw-opening reflex excitability was repetitively evaluated for 60 min at 30 min intervals.

### Application of Laser Irradiation, Local Anesthesia and Thermal Stimulation

Laser irradiation was applied to the palatal mucosa of the maxillary right first molar region (Figure 1B). To exclude the spread of laser irradiation, a silicon rubber plate made of impression paste (682864, YOSHIDA, Tokyo, Japan) was used to cover the contralateral periodontium. CO_2_ (OPELASER PRO, YOSHIDA, Tokyo, Japan) and diode (OPELASER Filio, YOSHIDA, Tokyo, Japan) lasers were used as described in Table 1 to obtain a consistent laser output range. The distance between the tip of the laser fiber and the tissue surface and the irradiation time were determined using the following formula:

$$\begin{array}{l} t\;\left(s\right)=\frac{A\times \left\{\pi \times {\left[\left(\frac{B}{2}\right)+x\times \tan\left(\frac{C}{2}\right)\right]}^{2}\right\}}{P} \end{array}$$

where *t* is the duration of irradiation (sec), *A* is the energy density (J/mm^2^), *B* is the diameter of the output fiber tip (mm), *C* is the angle (radians) of the laser, and *P* is the heat capacity (J/sec). Laser irradiation was carried out immediately after the first jaw-opening reflex evaluation in the intact and D1 groups (**Figure 4A**). In the preirradiation groups, laser irradiation was applied immediately after the application of orthodontic force, and then jaw-opening reflex excitability was measured after one (PI-D1), three (PI-D3), or seven (PI-D7) days C (**Figure 6A**).

**Table 1:** Laser irradiation parameters.

**Type of laser**	**CO_**2**_**	**Diode**
Output wavelength (nm)	10,600	808
Mean power output (W)	0.5	
Output mode	Continuous wave	
Irradiation time (sec)	15, 30 or 600	30 or 600
Distance (cm)^*^	20	3.2
Irradiation area (cm^2^)	1.67	
Type of guide laser	Diode laser	

* *From the tip of the laser fiber to the tissue surface*.

Local anesthesia was applied to the palatal side of the maxillary first molar mucosa. Ethyl aminobenzoate (0.02 g; Benzocaine Dental Jelly 20%, Bee Brand Medico Dental Co., Ltd., Osaka, Japan) was applied to the mucosal surface for surface anesthesia, and lidocaine (0.10 ml; xylocaine injection 2% with epinephrine, AstraZeneca, Osaka Japan) was injected into the mucosa for infiltration anesthesia. After 5 min, jaw-opening reflex excitability was evaluated (Figure 11A).

Thermal stimulation was applied using a custom-made heat probe (diameter: 3.5 mm; screw and copper wire). A constant current (1.2 A at 2 V) was supplied to the copper wire to warm the probe tip to 45°C. The probe was brought into contact with the palatal surface mucosa of the right maxillary first molar for 30 s (**Figures 9A**, **10A**).

### Temperature Measurement of the Mucosal Surface and Gingival Sulcus

The surface temperature of the laser-irradiated oral mucosa was measured with an infrared thermometer (IT-545 NH, HORIBA, Kyoto, Japan) in intact animals before and after each 30 s of laser irradiation. The temperature of the gingival sulcus was continuously measured by using a digital thermometer (BWT-100A00A, Bio Research Center, Aichi, Japan) with a temperature microprobe (diameter: 0.14 mm; IT-23, Physitemp, NJ, USA) and was stored with the EMG data (CED 1401 Plus) for offline analysis. The temperature microprobe was inserted into the palatal side of the gingival sulcus (2.0 mm).

### GFAP Immunostaining and Measurement of Neurons Encircled by GFAP-Immunoreactive (IR) Cells in the TGs

After the evaluation of jaw-opening reflex excitability, rats were administered an overdose of anesthetic and fixed via transcardial perfusion of isotonic saline followed by buffered 4% paraformaldehyde. The bilateral TG were removed from the animals in the intact, D1 (30 s of CO_2_ or diode laser irradiation) and PI-D1 (30 s of CO_2_ or diode laser irradiation) groups (*n* = 5 each). The removed tissue was kept in the same fixative solution for one additional day at 4°C and then kept in 0.01 M phosphate-buffered saline (PBS) containing 20% sucrose (w/v) for 12 h for cryoprotection. For cryosectioning, the specimens were embedded in Tissue Tek (Sakura Finetek Japan, Tokyo, Japan) and stored at −20°C. Ten-micron horizontal TG sections were obtained along the long axis. Every 15th section was thaw-mounted on MAS-GP glass microscope slides (Mastunami, Osaka, Japan) and dried overnight at room temperature. Four sections were chosen from each TG in each rat to be processed for GFAP immunohistochemistry. The sections were incubated with a mouse anti-GFAP monoclonal antibody (Millipore, Billerica, MA, USA) after dilution at a concentration of 1:800 in 0.01 M PBS containing 4% normal goat serum (NGS) and 0.3% Triton X-100 (Millipore, Sigma, St. Louis, MO, USA) overnight at 4°C. After rinsing with 0.01 M PBS, the sections were incubated in Alexa Fluor 568 anti-mouse IgG (1:200 in 0.01 M PBS; Invitrogen, Paisley, U.K.) for 2 h at room temperature. After rinsing with 0.01 M PBS, the sections were coverslipped in mounting medium (Thermo Fisher Scientific, Fremont, CA, USA), examined under a fluorescence microscope and analyzed with MIPAR Base (MIPAR, Worthington, OH, USA). No specific labeling was observed in the absence of the primary antibody. TG neurons with more than 2/3 of the perimeter surrounded by GFAP-IR cells were defined as TG neurons encircled with GFAP-IR cells. The number of TG neurons encircled with GFAP-IR cells was counted in each rat, and the relative number was calculated by the following formula: 100 × number of neurons encircled with GFAP-IR cells/total number of neurons (22).

### Measurement of ETM

In each animal in the PI (PI-D1, PI-D3, and PI-D7) and nonirradiated (D1, D3, and D7) groups, an impression of the maxillary dental arch was obtained with silicone impression paste before spring application (pre-cast) and after jaw-opening reflex evaluation (post-cast) to obtain dental casts. In both casts, the distance between the mesial surface of the maxillary first molar and the distal surface of the seconds molar was measured by a Vernier caliper (resolution: 0.01 mm; model AD-10AX; Mitutoyo Corporation, Kanagawa, Japan), and the difference was defined as the amount of tooth movement (**Figure 8A**). The measurement was carried out by two blinded investigators (3 times each) for each cast, and the mean value was calculated (21).

### Data Analysis and Expression

The right-side threshold for inducing the jaw-opening reflex and related EMG parameters were standardized to those obtained from left-side stimulation. For example, each threshold (%) is expressed as the threshold for inducing the jaw-opening reflex on the left and right sides (μA)/threshold for inducing the jaw-opening reflex on the left side that was obtained before treatment (e.g., irradiation, thermal stimulation and local anesthesia) (μA). Because the AUC was variable across animals, the suprathreshold intensity-induced AUC was standardized to that obtained by threshold stimulation in each animal. Data are expressed as the mean ± SE. One-way ANOVA or two-way ANOVA was performed, followed by the Bonferroni *post hoc* test or Dunnett's *post hoc* test. Student's *t*-test or paired *t*-test was used for comparisons between two groups, as appropriate. A *p* < 0.05 was considered to be significant. OriginPro 2018 (OriginLab, Northampton, MA, USA) was used for all statistical analyses.

## Results

### Influence of Laser Irradiation on Jaw-Opening Reflex Excitability and Mucosal Temperature in Intact Animals

**Figure 2:**
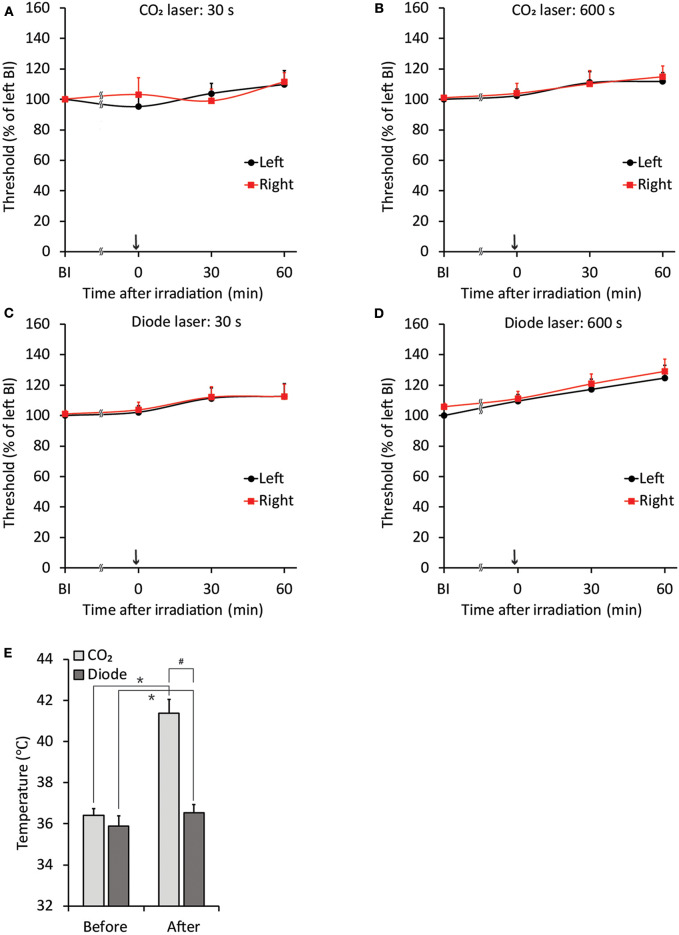
Stability of jaw-opening reflex excitability and temperature alterations after laser irradiation in rats without application of orthodontic force. **(A–D)** There were no significant differences between the left- and right-side thresholds for inducing the jaw-opening reflex (BI). The application of CO_2_ (**A**: 30 s, **B**: 600 s) or diode (**C**: 30 s, **D**: 600 s) laser irradiation did not alter jaw-opening reflex excitability. **(E)** CO_2_ laser irradiation significantly increased the surface temperature of the right maxillary first molar gingiva. BI: before laser irradiation. Arrow: laser irradiation. Before: before irradiation. After: after irradiation. Paired *t*-test, ^*^p < 0.05, Student's *t*-test, ^#^*p* < 0.05.

**Figure 3:**
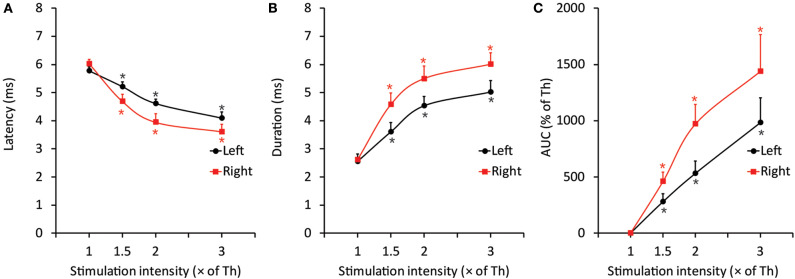
Representative data of EMG features induced by threshold and suprathreshold stimulation in the intact group after 30 s of CO_2_ laser irradiation to elucidate the less invasive effects of laser irradiation on the jaw-opening reflex system. **(A–C)** The excitability of all three EMG parameters was increased in a stimulation intensity-dependent manner and was observed as a significant decrease in latency and a significant increase in duration and AUC. The application of orthodontic force or laser irradiation did not alter the relationship between stimulation intensity and EMG excitability. Th, threshold; AUC, area under the curve. One-way ANOVA, *p* < 0.05. Dunnett's *post hoc* test, ^*^p < 0.05 vs. “threshold”.

**Figure 4:**
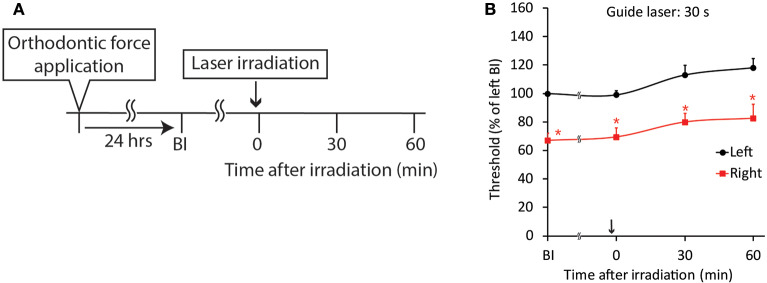
Effect of guide laser irradiation on orthodontic treatment-induced excitation of the jaw-opening reflex. **(A)** Experimental procedure for the application of orthodontic force, guide laser irradiation and the evaluation of jaw-opening reflex excitability. **(B)** One day of orthodontic force application significantly reduced the right-side threshold for inducing the jaw-opening reflex, and guide laser irradiation of the maxillary first molar region for 30 s failed to alter the reduction in the threshold for 60 min. BI: before laser irradiation. Arrow: laser irradiation. Left vs. right: two-way ANOVA, side: *p* < 0.05, time: *p* < 0.05, side × time: *p* < 0.05; Bonferroni *post hoc* test, ^*^p < 0.05 vs. “BI of left”.

**Figure 5:**
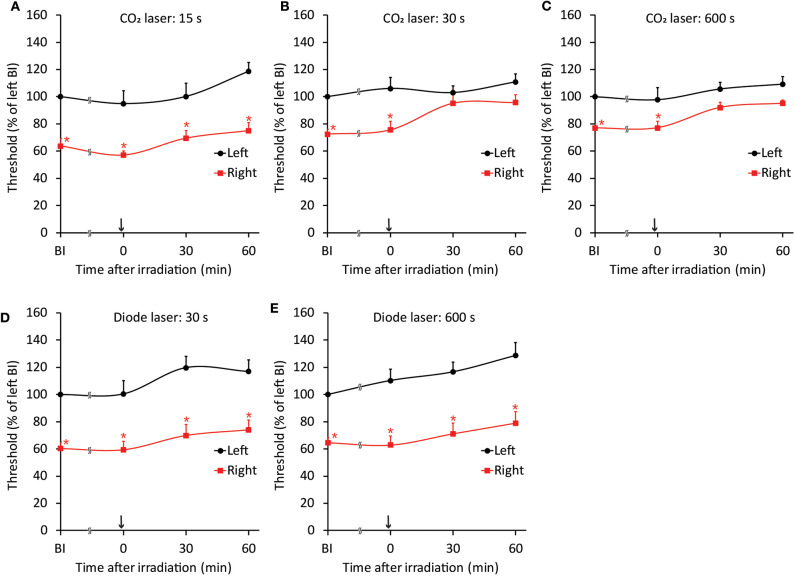
Effects of CO_2_ or diode laser irradiation on orthodontic treatment-induced excitation of the jaw-opening reflex. **(A–C)** One day of orthodontic force application significantly reduced the right-side threshold for inducing the jaw-opening reflex before irradiation. CO_2_ laser irradiation of the maxillary first molar region for 15 s **(A)** failed to alter jaw-opening reflex excitability. However, 30 **(B)** and 600 **(C)** s of CO_2_ laser irradiation significantly increased the right-side threshold for inducing the jaw-opening reflex. **(D,E)** Thirty seconds **(D)** or 600 s **(E)** of diode laser irradiation failed to increase the right-side threshold for inducing the jaw-opening reflex. BI: before irradiation. Arrow: laser irradiation. Two-way ANOVA, side: *p* < 0.05, time: *p* < 0.05, side × time: *p* < 0.05; Dunnett's *post hoc* test, ^*^p < 0.05: vs. “BI of left”.

**Figure 6:**
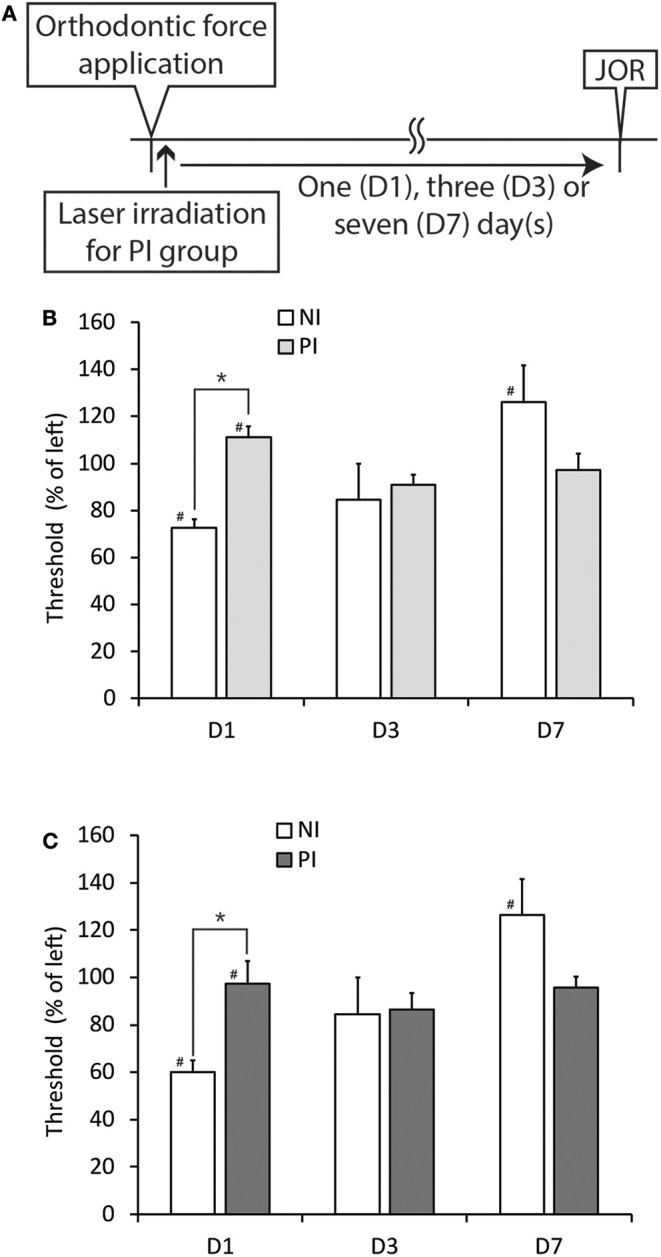
Effects of CO_2_ or diode laser irradiation immediately after the application of orthodontic force (preirradiation) on the development of orthodontic treatment-induced excitation of the jaw-opening reflex. **(A)** Experimental procedure for the application of orthodontic force, laser irradiation and the evaluation of jaw-opening reflex excitability. **(B)** Thirty seconds of CO_2_ laser irradiation significantly increased the right-side threshold for inducing the jaw-opening reflex on D1, but this effect was not observed at three or seven days after PI (D3 and D7). **(C)** Thirty seconds of diode laser irradiation significantly increased the right-side threshold for inducing the jaw-opening reflex on D1, but this effect was not observed at 3 or 7 days after PI (D3 and D7). NI, non-irradiation; PI, preirradiation; JOR, evaluation of jaw-opening reflex excitability. Student's *t*-test, ^*^p < 0.05: NI vs. PI. Paired *t*-test, ^#^*p* < 0.05: left vs. right in the same group.

**Figure 7:**
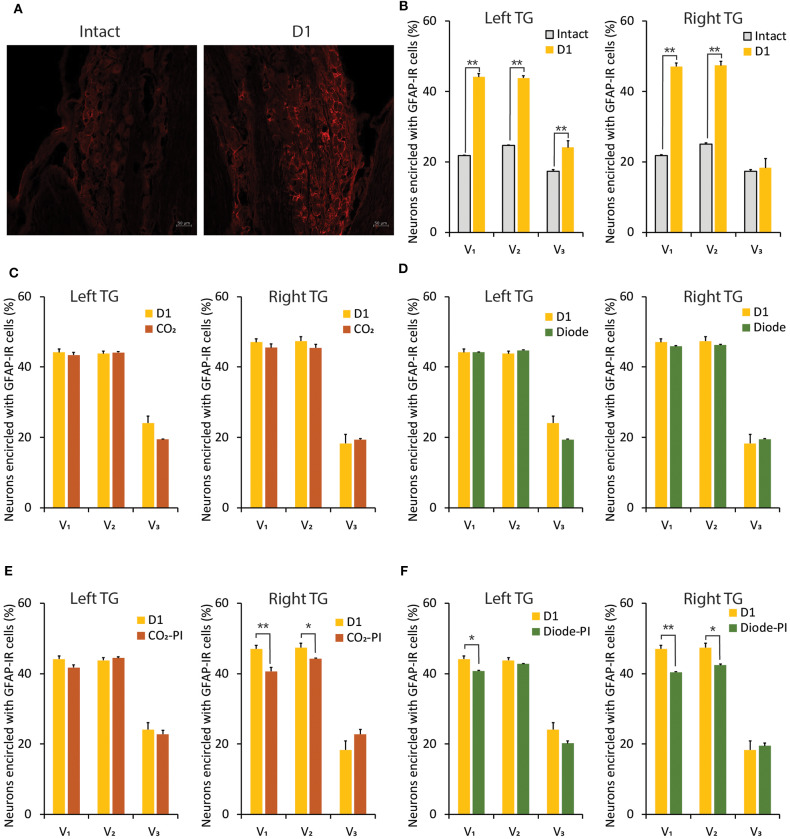
Alterations in GFAP-IR cells expression in the TG induced by orthodontic treatment and laser irradiation. **(A)** Immunohistochemical photomicrographs of TG neurons in the V_2_ branch region encircled with GFAP-IR cells in intact (left) and D1 (right) animals. Scale bar: 50 mm. **(B)** Comparison of the number of TG neurons encircled with GFAP-IR cells in the V_1_, V_2_, and V_3_ branch regions between intact and D1 animals. **(C,D)** Thirty seconds of CO_2_ **(C)** or diode **(D)** laser irradiation on D1 failed to alter the number of TG neurons encircled with GFAP-IR cells in the V_1_, V_2_, and V_3_ branch regions. **(E,F)** Thirty seconds of CO_2_ **(E)** or diode **(F)** laser irradiation immediately after orthodontic force application (preirradiation) significantly altered the number of TG neurons encircled with GFAP-IR cells in the V_1_, V_2_, and V_3_ branch regions. PI, preirradiation. Student's *t*-test, ^**^p < 0.01, ^*^p < 0.05.

**Figure 8:**
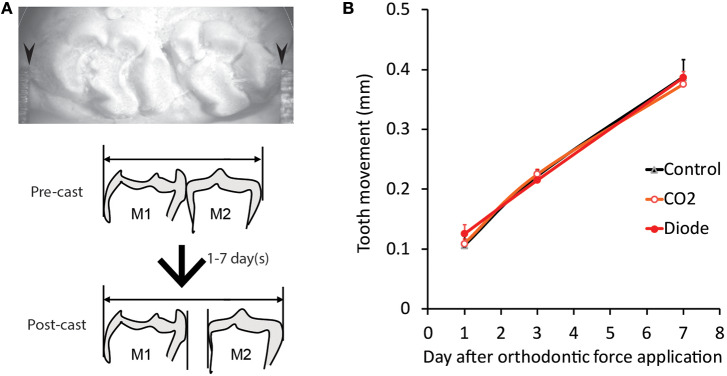
Effects of laser irradiation on orthodontic force-induced ETM. **(A)** Representative photomicrograph of tooth movement measurement in the plaster model (post-cast) (top) and schematic drawing of the lateral view of the pre- and post-casts of the molars (bottom). **(B)** The distance of tooth movement at one (D1), three (D3) and seven (D7) days after the application of orthodontic force in each experimental group. Arrowheads: contact point between the molars of the plaster model and the Vernier caliper.

**Figure 9:**
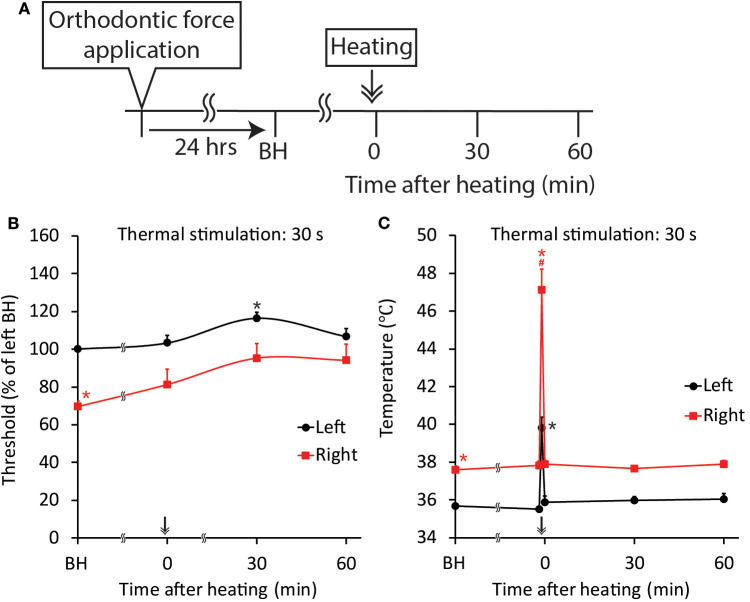
Effect of thermal stimulation on orthodontic treatment-induced excitation of the jaw-opening reflex. **(A)** Experimental procedure for the application of orthodontic force, thermal stimulation and the evaluation of jaw-opening reflex excitability. **(B)** Thermal stimulation (30 s, 45°C) significantly increased the right-side threshold for inducing the jaw-opening reflex. **(C)** Thermal stimulation (30 s, 45°C) significantly increased the gingival sulcus temperature on both sides. BH, before heating (thermal stimulation). Arrow & Heating: thermal stimulation. Two-way ANOVA, side: *p* < 0.05, time: *p* < 0.05, side × time: *p* < 0.05; Dunnett's *post hoc* test, ^*^p < 0.05: vs. “BH of left,” ^#^*p* < 0.05: vs. “BH of right”.

**Figure 10:**
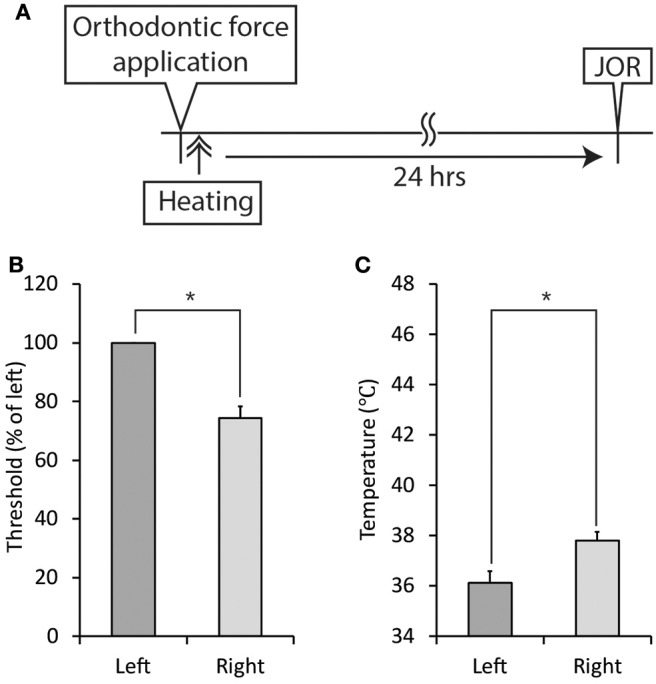
Effect of thermal stimulation immediately after the application of orthodontic force on the development of orthodontic treatment-induced excitation of the jaw-opening reflex. **(A)** Experimental procedure for the application of orthodontic force, thermal stimulation, and the evaluation of jaw-opening reflex excitability. **(B)** Thermal stimulation (30 s, 45°C) did not alter the right-side threshold for inducing the jaw-opening reflex on D1. **(C)** Thermal stimulation (30 s, 45°C) did not alter the temperature of the right molar gingival sulcus on D1. Heating: thermal stimulation. JOR, evaluation of jaw-opening reflex excitability. Paired *t*-test, ^*^p < 0.05.

**Figure 11:**
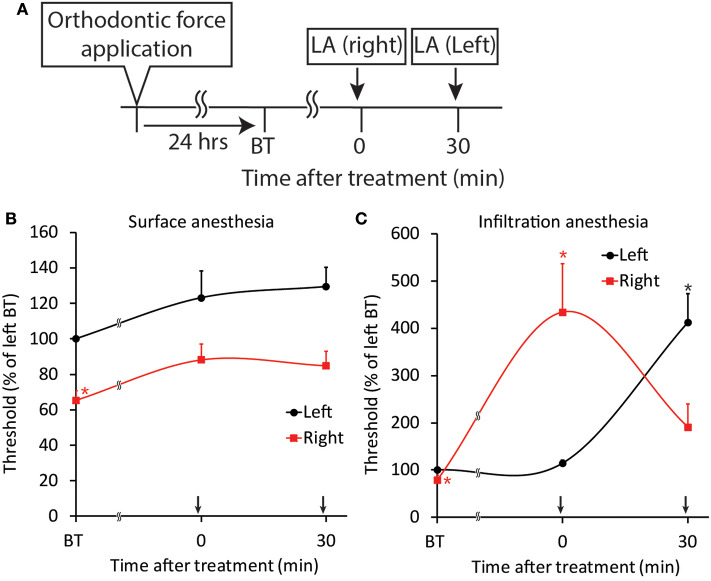
Effect of local anesthesia on orthodontic treatment-induced excitation of the jaw-opening reflex. **(A)** Experimental procedure for the application of orthodontic force, local anesthesia, and the evaluation of jaw-opening reflex excitability. **(B)** Surface anesthesia applied to the right maxillary first molar region significantly increased the right-side threshold for inducing the jaw-opening reflex. **(C)** Infiltration anesthesia applied to the right maxillary first molar region increased the right-side threshold for inducing the jaw-opening reflex. LA, local anesthesia; BT, before treatment (local anesthesia). Arrow: local anesthesia. Two-way ANOVA, side: *p* < 0.05, time: *p* < 0.05, side × time: *p* < 0.05; Dunnett's *post hoc* test, ^*^p < 0.05: vs. “BT of left”.

The threshold intensity for inducing the jaw-opening reflex varied among intact animals; however, the threshold intensity was quite stable within each animal, and there were no significant differences between left- and right-side stimulation (Figures 2A–D: BI). After CO_2_ laser irradiation of the right maxillary first molar region, the threshold slightly increased (30 s: left: <9.7 ± 9.0%, right: <11.1 ± 6.3%; 600 s: left: <11.9 ± 5.8%, right: <13.8 ± 6.8%) for 60 min, and there were no significant differences between left- and right-side stimulation (Figures 2A,B, 0–60 min). Similar alterations were observed in the animals treated with diode laser irradiation (30 s: left: <12.5 ± 8.4%, right: <11.4 ± 7.9%; 600 s: left: <24.7 ± 7.4%, right: <23.5 ± 9.4%) (Figures 2C,D: 0–60 min). After 30 s of irradiation, the CO_2_ laser significantly (*p* < 0.05) increased the temperature of the right maxillary mucosa (Figure 2E), while the diode laser did not alter the temperature significantly; there was a significant (*p* < 0.05) difference between CO_2_ and diode laser irradiation. Significant alterations in jaw-opening reflex-related EMG parameters were observed as stimulation-dependent reductions in latency and increases in duration and AUC (Figure 3). These alterations of EMG parameters were similarly observed on left- and right-side stimulation in all experimental groups, including the ETM groups (not shown). Figure 3 shows the alterations observed in intact animals that received 30 s of CO_2_ laser irradiation.

### Influence of Laser Irradiation on Jaw-Opening Reflex Excitability in the ETM Groups

Because both the CO_2_ and diode lasers were equipped with a guide laser (Table 1) to indicate the approximate range of irradiation, the effect of guide laser irradiation on jaw-opening reflex excitability was evaluated in D1 animals. One day of orthodontic force application to the right maxillary first molar significantly (*p* < 0.05) reduced the threshold for inducing the jaw-opening reflex on right-side stimulation compared with left-side stimulation (Figure 4B: BI). Thirty seconds of guide laser irradiation did not alter the significant reduction in the right-side threshold until 60 min after irradiation (Figure 4B: 0–60 min). Both 30 and 600 s of CO_2_ laser irradiation significantly (*p* < 0.05) increased the right-side threshold at 30 min after irradiation. The analgesic effect lasted for 60 min (Figures 5B,C: 30–60 min); however, 15 s of CO_2_ laser irradiation failed to alter the right-side threshold. The threshold for inducing the jaw-opening reflex on left-side stimulation was not altered by 15–600 s of CO_2_ laser irradiation (Figures 5A–C). Thirty and 600 s of diode laser irradiation did not alter the threshold for inducing the jaw-opening reflex on either left- and right-side stimulation (Figures 5D,E). Prolonged application of orthodontic force induced temporal alterations in jaw-opening reflex excitability. The reduction in the right-side threshold for inducing the jaw-opening reflex was almost recovered on D3, and the threshold exceeded that on the left side on D7 (Figures 6B,C). The effects of laser irradiation immediately after orthodontic force application on the temporal alterations in jaw-opening reflex excitability were investigated in the preirradiated groups. Compared with the nonirradiated D1 group, preirradiation (30 s of CO_2_ laser) significantly (*p* < 0.05) increased the threshold for inducing the jaw-opening reflex on right-side stimulation (Figure 6B) on D1. On D3 and D7, the threshold for inducing the jaw-opening reflex was not significantly different from that in the non-irradiated groups. Interestingly, preirradiation prohibited the increase in the threshold for inducing the jaw-opening reflex on D7. Similar effects were observed in animals irradiated by a diode laser for 30 s immediately after the application of orthodontic force (Figure 6C). Compared with that in the non-irradiated D1 group, the threshold for inducing the jaw-opening reflex was significantly (*p* < 0.05) increased in the preirradiated group, and subsequent temporal alterations in jaw-opening reflex excitability were almost the same as those observed after preirradiation by a CO_2_ laser (Figures 6B,C).

### Alterations in GFAP-IR Cells Expression Induced by Orthodontic Force Application and Laser Irradiation

In comparison with the intact control, 1-day of orthodontic force application significantly (*p* < 0.01) increased the number of neurons encircled with GFAP-IR cells in the V_1_ and V_2_ branch regions of the bilateral TGs (Figures 7A,B). On the left but not the right side, the expression of GFAP-IR cells in the TG was also significantly (*p* < 0.01) increased in the V_3_ branch region. Irradiation by a CO_2_ or diode laser at 1 day after the application of orthodontic force failed to alter the number of TG neurons encircled with GFAP-IR cells (Figures 7C,D). On the other hand, preirradiation by either laser significantly reduced the number of neurons encircled with GFAP-IR cells in the V_1_ (*P* < 0.01) and V_2_ (*p* < 0.05) branch regions of the right TG. In addition, preirradiation with a diode laser significantly (*p* < 0.05) reduced the expression of GFAP-IR cells in the V_1_ branch region of the left TG (Figures 7E,F).

### Effect of Laser Irradiation on the Distance of ETM

Without laser irradiation, the application of orthodontic force induced a temporal increase in the distance of tooth movement, as previously described (21). Thirty s of irradiation by a CO_2_ or diode laser did not alter the distance of tooth movement at any of the observation timepoints (D1, D3, or D7) (Figure 8B).

### Effect of Thermal Stimulation on Jaw-Opening Reflex Excitability in the ETM Groups

Thermal stimulation (30 s, 45°C) of the palatal mucosa of the right maxillary first molar significantly (*p* < 0.05) increased the right-side threshold for inducing the jaw-opening reflex immediately after thermal stimulation, and the increase in the threshold was sustained until the termination of observation (Figure 9B). Thermal stimulation of the right molar region also increased the left-side threshold for inducing the jaw-opening reflex 30 min later. One day of orthodontic force application significantly (*p* < 0.05) increased the temperature of the gingival sulcus of the right maxillary first molar compared with that of the contralateral molar (Figure 9C: BH). Thermal stimulation of the right-side molar region significantly (*p* < 0.05) and promptly increased the temperature of the gingival sulcus of the right molar, and it returned to the previous level immediately after the termination of stimulation. Similarly, but to a much lesser extent, temporal alterations in the temperature of the gingival sulcus of the left molar were observed. Thermal stimulation immediately after the application of orthodontic force failed to alter the right-side threshold for inducing the jaw-opening reflex on D1 (Figure 10B). In those animals, a significant (*p* < 0.05) difference in the temperature of the gingival sulcus was observed between the left and right molars (Figure 10C).

### Effect of Local Anesthesia on Jaw-Opening Reflex Excitability in the ETM Groups

Both surface and infiltration anesthesia of the palatal mucosa of the right maxillary first molar significantly (*p* < 0.05) increased the right-side threshold for inducing the jaw-opening reflex, and the increase in the threshold was sustained until the termination of observation (Figures 11B,C). Although surface anesthesia of the left molar region did not alter the left-side threshold for inducing the jaw-opening reflex, infiltration anesthesia of the left molar region significantly (*p* < 0.05) increased the left-side threshold for inducing the jaw-opening reflex. The intensity of the left- and right-side thresholds after infiltration anesthesia were similar.

## Discussion

In this study, the beneficial effects of laser irradiation by common dental lasers using different light sources on the orthodontic pain-related reflex and ETM were examined. In this animal model, the threshold for inducing the jaw-opening reflex was significantly reduced on the right side, which was subjected to orthodontic force, compared to the left side, which remained intact. These findings are consistent with those of previous studies reporting that trigeminal (e.g., pulp and temporomandibular joint) noxious stimulation increases jaw-opening reflex excitability (27–29) and that orthodontic force-induced inflammation in the periodontium (e.g., the periodontal ligaments and alveolar bone) plays a crucial role in the excitability of the jaw-opening reflex (21). In addition, it has been confirmed previously that the application of an orthodontic apparatus without spring elongation did not alter the excitability of the jaw-opening reflex (21).

Two types of lasers are available for therapeutic usage: one is characterized by long wavelengths (1,064–10,600 nm) and is absorbed in surface tissue (e.g., CO_2_ and Nd:YAG lasers); the other is characterized by short wavelengths (633–890 nm) and penetrates tissue (e.g., diode lasers) (16). The analgesic effect of laser irradiation has been investigated in humans and animals; however, the results of those investigations are not consistent. In humans, sufficient and insufficient effects of LLLT by either CO_2_ (11, 15, 19) or diode laser (12, 20) irradiation have been reported. In animals, although CO_2_ lasers have been used for noxious stimulation (30), analgesic effects of other lasers with long wavelengths have been reported (13, 31). To exclude invasive effects and to evaluate the beneficial effects of CO_2_ laser irradiation, an appropriate irradiation distance between the tip of the laser fiber and the tissue surface had to be determined. We set the irradiation distance for the CO_2_ laser as 20 cm away from the tissue surface because irradiation at this distance did not induce a pain sensation in human skin. Based on the irradiation distance of the CO_2_ laser, the distance for the diode laser was calculated as 3.2 cm to obtain a consistent irradiation range. Under these conditions, neither CO_2_ nor diode laser irradiation altered jaw-opening reflex excitability in intact animals, and a stimulation intensity-dependent increase in EMG parameters was observed, as reported in a previous study (21). These observations indicated that laser irradiation by neither laser induced invasive effects on trigeminal sensation or muscle (e.g., anterior digastric and masseter) function under the current conditions. The wavelength of the CO_2_ laser is in the far-infrared range (>1,200 nm). It has been reported that such lasers penetrate tissue only 20 μm (14) and are then absorbed by the moisture of the tissue, increasing the temperature (17). On the other hand, the wavelength of the diode laser is in the near-infrared range (700–1,200 nm) (14), and these lasers penetrate tissue 4 mm and are not absorbed by tissue moisture (17). Differences in the thermal effects of CO_2_ and diode lasers may play a critical role in the acute analgesic effect in this model because the diode-sourced guide laser did not show an analgesic effect. Indeed, an increase in the local circulation of the CO_2_ laser-irradiated area has been reported (11, 32), which may increase the clearance of inflammatory mediators in inflamed tissue. In humans, similar acute analgesic effects of CO_2_ laser irradiation on tissue with mucosal disease (e.g., pemphigus vulgaris and aphthous stomatitis) have been reported (15, 33). Zand et al. applied a CO_2_ laser through a thick layer of transparent, high-water-content gel, and there were no significant alterations in the mucosal temperature after irradiation (33). This result suggests the importance of evaluating the local anesthetic effect of CO_2_ laser irradiation. In the present study, CO_2_ but not diode laser irradiation, thermal stimulation and surface anesthesia increased the right-side threshold for inducing the jaw-opening reflex similarly on D1. From these results, the involvement of both temperature alterations and surface anesthetic effects in the acute analgesic effect of CO_2_ laser irradiation was evident. Since infiltration anesthesia increased the threshold for inducing the jaw-opening reflex to a much higher level (400% of BI) on both sides, the surface anesthetic effect of the CO_2_ laser might be related to the absorption rate of the longer laser wavelength. Moreover, the involvement of transient receptor potential vanilloid 1 (TRPV1) and transient receptor potential ankyrin 1 (TRPA1) in the acute analgesic effect of CO_2_ laser irradiation has been suggested (34, 35). TRPV1V is a highly temperature-sensitive (>42°C) non-selective cation ion channel (36, 37); however, it is rapidly (on the ms-to-s time scale) desensitized, and the molecular basis of the heat desensitization of TRPV1 has recently been reported (38). Moreover, the inhibition of acid-sensing ion channels by TRPV1 activation has also been reported (39). On the other hand, TRPA1 is a lower temperature-sensitive (<20°C) non-selective cation ion channel and is also involved in pain sensation (37). Although the activating temperature of TRPA1 is different from that of TRPV1, agonist-activated TRPA1 is suppressed by warmth (>40°C) (35). Since both CO_2_ laser irradiation and thermal stimulation rapidly increased the peripheral temperature in the present study in the range of the heat desensitization of TRPV1 and TRPA1, further investigation of the crucial role of TRPV1 and TRPA1 in orthodontic force-induced pain is emphasized. Nevertheless, effects other than those described above are likely involved in the preventive effects of both lasers on orthodontic force-induced excitation of the jaw-opening reflex in the preirradiation groups. Indeed, thermal stimulation immediately after orthodontic force application failed to alter jaw-opening reflex excitability on D1. Diode laser irradiation has been used for photodynamic therapy (17), and biologically relevant photochemical reactions are dependent on the generation of reactive oxygen species (ROS) (40, 41) and singlet oxygen (^1^O_2_) (42). One potential cause of the preventive effects of the diode laser on orthodontic force-induced excitation of the jaw-opening reflex in this study is ROS because laser irradiation at a wavelength <800 nm is required to produce a therapeutic concentration of ^1^O_2_ (17). Moreover, a recent report on the enhancement of ROS generation by CO_2_ laser irradiation (43) suggested that ROS were also involved in the preventive effects of the CO_2_ laser irradiation on orthodontic force-induced excitation of the jaw-opening reflex. ROS are involved in two different aspects of biological function: redox biology and oxidative stress (44). To elucidate whether ROS have beneficial effects on orthodontic force-induced pain, further investigation into the functional relationship between laser irradiation-induced ROS generation and the production of inflammatory chemical mediators is required. Although the molecular basis of the preventive effects of both lasers on orthodontic force-induced excitation of the jaw-opening reflex is still unclear, the modulation of inflammation might play an important role. The increases in the gingival sulcus temperature and the number of TG neurons encircled with GFAP-IR cells induced by the application of orthodontic force on D1 indicated the presence of inflammation around the treated molar. An inhibitory effect of anti-inflammatory drugs, such as aspirin, on orthodontic treatment-induced excitation of the jaw-opening reflex has been reported (21). In the present study, the significant reduction in the number of TG neurons encircled with GFAP-IR cells was associated with a significant decrease in jaw-opening reflex excitability. These results suggest that the preventive effects of both lasers on orthodontic force-induced excitation of the jaw-opening reflex are associated with the attenuation of inflammation but do not alter tooth movement (see below). Nerve injury induces the expression of GFAP in dorsal root ganglionic SGCs within 4 h, and this effect is reduced in 3 weeks (45). This temporal pattern of GFAP expression is consistent with our results, such as the slight increase in the number of TG neurons encircled with GFAP-IR cells in intact animals compared with the results of a previous study (22), because those animals underwent the orofacial surgical procedure and the evaluation of jaw-opening reflex excitability before the histochemical procedure (e.g., perfusion). Furthermore, an attenuating effect of irradiation with either laser on GFAP expression is hypothesized to occur because preirradiation inhibits the increase in the threshold for inducing the jaw-opening reflex on D7. The alterations in GFAP-IR cell expression in the TG are related to the expression of referred pain (46). The application of orthodontic force significantly increased the number of neurons encircled with GFAP-IR cells in not only the V_2_ but also the V_1_ and V_3_ branch regions in the bilateral TGs. SGCs are connected by gap junctions; thus, excitatory information might spread from the V_2_ branch region to other regions in the unilateral TG. It has been documented that unilateral noxious stimulation of the orofacial region increased neural activity in the bilateral trigeminal spinal subnucleus caudalis (Vc), upper cervical spinal cord (C1/C2), and paratrigeminal nucleus (Pa5) (26), which are thought to organize somatotopical information related to orofacial pain. This result suggests the contribution of those nuclei to the increase in GFAP-IR cells in the contralateral TG.

The ETM was measured to assess another effect of laser irradiation. Recently, a significant increase in tooth movement mediated by irradiation with 660 or 830 nm lasers was reported (47). Laser irradiation also significantly increased the expression of interleukin-1β (IL-1β), receptor activator of nuclear factor κ-B ligand (RANKL), and osteoprotegerin (OPG) and the activity of osteoclasts in a wavelength-dependent manner. Yang et al. applied laser irradiation almost every day for 1 week, and a significant increase in ETM was observed after 14 days. In the present study, neither CO_2_ laser nor diode laser irradiation increased ETM. This inconsistency may be related to the irradiation procedure (e.g., frequency and period) and the observation period. However, this difference raises the possibility that the biological effect of short-wavelength lasers on the synthesis of IL-1β, RANKL and OPG appears rapidly but does not last long enough to influence tooth movement.

## Data Availability Statement

All datasets generated for this study are included in the article/supplementary material.

## Ethics Statement

The animal study was reviewed and approved by Animal Experimentation Committee of Meikai University School of Dentistry.

## Author Contributions

All authors had full access to all of the data in the present study and take responsibility for the integrity of the data and the accuracy of the data analysis. KA, NS, and TT: study concept and design and drafting of the manuscript. TT, MY, NH, AS, and KA: data acquisition. TT, MY, NH, NS, and KA: data analysis and interpretation. TT, MY, NS, NH, AS, and KA: critical revision of the manuscript. KA: study supervision.

## Conflict of Interest

The authors declare that the research was conducted in the absence of any commercial or financial relationships that could be construed as a potential conflict of interest.
